# Moving beyond the tracer conditions: positioning childhood cancer within the scale-up of PEN-Plus in Africa

**DOI:** 10.3389/fonc.2026.2006160

**Published:** 2026-10-30

**Authors:** Martin Heine, Choolwe Jacobs, Larissa Klootwijk, Harrison Andeko, Ombeni Idassi, Paul Kamfwa, Edna Majaliwa, Ana O. Mocumbi, Bavin Mulenga, Annie C. Mwila, Judith Namuyonga, Laylah Ryklief, John Ssentamu, Isaac Ssinabulya, Fastone Goma

**Affiliations:** 1 Princess Máxima Center for Pediatric Oncology, Utrecht, Netherlands; 2 School of Public Health, University of Zambia, Lusaka, Zambia; 3 NCD Alliance, Nairobi, Kenya; 4 St. Jude Children’s Hospital, Memphis, TN, United States; 5 Ministry of Health, Lusaka, Zambia; 6 Muhimbili National Hospital, Dar-es-Salaam, Tanzania; 7 NCDi Poverty Network, Maputo, Mozambique; 8 Universidade Eduardo Mondlane, Maputo, Mozambique; 9 UNICEF, Lusaka, Zambia; 10 Uganda Heart Institute, Kampala, Uganda; 11 Research Centre for Adolescent and Child Health (REACH), University of Cape Town, Cape Town, South Africa; 12 Partners in Health, Monrovia, Liberia; 13 Center for Infectious Disease Research in Zambia, Lusaka, Zambia

**Keywords:** Africa, childhood cancer, low-to-middle income countries, non-communicable diseases, pediatric care, PEN-Plus

## Abstract

The 3rd International Conference on PEN-Plus in Africa (ICPPA) reaffirmed PEN-Plus’s potential to decentralise care for severe, complex non-communicable diseases and injuries among underserved populations, particularly children and adolescents. Although childhood cancer was recognised as a sentinel condition in the original Lancet Commission report, it has not yet been fully embraced within PEN-Plus implementation. This gap is striking given that nearly 90% of the over 100–000 children who develop cancer annually in sub-Saharan Africa die, largely as a result of underdiagnosis and late-stage presentation. First-level referral hospitals could therefore help bridge primary and tertiary care, improving timely diagnosis, early treatment, and adherence. Accordingly, the next phase of PEN-Plus should be defined not only by expanding coverage to improve access to care, but also by strengthening equitable, child-centred, and integrated service delivery within national primary health care systems and communities – including extending comprehensive services to other orphaned conditions such as childhood cancer.

## 1 Introduction

The 3rd International Conference on PEN-Plus in Africa (ICPPA) recently reaffirmed PEN-Plus’s potential for decentralizing care for severe, complex non-communicable diseases and injuries (NCDIs) among underserved populations in the African Region, particularly children and adolescents. PEN-Plus complements the WHO Package of Essential Noncommunicable Disease Interventions (WHO PEN), which is largely focused on adult conditions managed at the primary care level, by extending decentralized outpatient services and integrated case management to severe childhood-onset NCDIs (1).

Severe childhood-onset NCDIs result in more than 20 years of life lost per death and include a diverse group of childhood-onset NCDs (sickle cell disease, epilepsy, asthma, chronic kidney disease, rheumatic heart disease, type 1 diabetes), congenital anomalies, cancers, and acute abdominal conditions (1). The PEN-Plus model of care - comprising standardized programmatic protocols, a task-sharing approach involving training and mentorship of non-specialist clinicians, and integrated patient-centred support (1, 2), has to date, been formally adopted by 29 partner countries in Africa, of which 15 have progressed to active implementation at first-referral hospitals for a subset of severe childhood-onset NCDIs, primarily type 1 diabetes, sickle cell disease, and childhood heart conditions. The pace and scope of implementation have varied across settings, shaped by the complexity of care required for specific conditions and the relative burden of childhood-onset NCDIs locally, reflecting the context-specific adaptations during scale-up. This process has yielded, to date, ≥120 operational PEN-Plus clinics and the training of more than 17,000 health care workers. Functioning as pivotal referral conduits within the health system, these clinics operate bidirectionally: referring patients to tertiary-level facilities for diagnostic confirmation and treatment initiation, while referring clinically stable patients to primary care facilities for continued management and monitoring. The effectiveness of this model was evidenced by unreported data presented at ICPPA (2026) from 16 clinics across nine countries, in which nearly 5,000 paediatric cases - 51% representing new diagnoses - were retained in care, achieving survival probabilities of 90% for sickle cell disease (SCD) and type 1 diabetes (T1D), and 80% for cardiac conditions.

This perspective reflects our collective insights as co-authors and participants in a roundtable discussion convened in parallel to ICPPA, in Dar es Salaam, Tanzania, on Tuesday, 23 June 2026, with the aim of sharing views on the challenges and opportunities in sustaining the paediatric focus of PEN-Plus during its implementation and scale-up. Participants in the roundtable were purposively invited, drawing on the ICPPA public agenda and existing professional networks, with the aim of ensuring a diversity of perspectives and experiences. Collectively, participants brought expertise in childhood-onset NCDs and/or PEN-Plus implementation across eight African countries, as well as broader regional experience through multinational partnerships, including the NCDI Poverty Network. The group also represented a range of professional perspectives, spanning program implementation, policy, academia, and clinical practice.

Four guiding questions were used to frame the discussion – which did not intend to reach consensus rather to elicit diverse perspectives across diverse experiences:

- How are pediatric services currently being included within PEN-Plus implementation in your setting or across the region?
- What are the main challenges to sustaining pediatric services within PEN-Plus?
- What has helped, or could help, pediatric services remain visible and sustainable within PEN-Plus?
- What should be done over the next 3 to 5 years to ensure pediatric services are not “crowded out or deprioritized” as PEN-Plus scales up?

## 2 Key implementation challenges and opportunities

### 2.1 Integrating childhood cancer care into the PEN-Plus framework

However, despite these success stories, one evident gap remains in childhood cancer; recognized as a sentinel paediatric condition within the original Lancet Commission’ report (3), widely recognized for its burden of disease and broader impact on health and well-being for both patient and support system, though not fully embraced within the implementation of PEN-Plus. Modelled estimates suggest that more than 100,000 children develop cancer annually in sub-Saharan Africa, with mortality remaining disproportionately high (4, 5). However, empirical data on the true burden are relatively scarce, in part, because many children (estimated at 53%) with cancer may never receive a diagnosis or reach specialist care (4, 5). Illustrating this diagnostic gap, a retrospective study from Bungoma County in western Kenya found that only 13 children per year were referred with cancer to the regional tertiary centre between 2010 and 2016, representing 9% of an estimated 140 incident cases annually in the county; among those reaching care, the median total diagnostic and treatment delay was 108 days and event-free survival was 21% (6). This case example is illustrative for the substantial underdiagnosis, delayed access, and low survival rates for childhood cancer care experienced across the region (7–12). For children who are diagnosed, poor outcomes are further compounded by advanced disease at presentation, treatment abandonment, treatment-related toxicities, and comorbidities such as malnutrition (13, 14).

There is a critical role for first-level referral hospitals to bridge the gap between primary care and tertiary, specialized services in addressing issues of timely diagnosis, early treatment and enhanced treatment adherence. Evidence to this effect from Kenya highlights that virtual, system-wide, sensitization towards childhood cancer increased detection by 33% (15). The ratification of PEN-Plus by 47 countries in the African region (1–3), provides a timely vehicle to strengthen childhood cancer health systems at scale.

To address inequities in childhood cancer survival, the WHO launched the Global Initiative for Childhood Cancer (GICC), whose flagship framework, CureAll, is structured around four pillars - centres of excellence, universal health coverage, regimens and roadmaps, and evaluation and monitoring - underpinned by three cross-cutting enablers: advocacy, leveraged financing, and linked governance (16). The CureAll architecture maps closely onto that of PEN-Plus: decentralized, specialist-supervised care hubs correspond to centres of excellence; integration into national benefit packages and insurance schemes reflects universal health coverage; standardized clinical protocols align with regimens/roadmaps; and facility-readiness monitoring conducted through the NCDI Poverty Network constitutes the evaluation component, with the Network itself functioning as the enabler layer. Integrating these two widely adopted strategic frameworks could offer a contemporary structure for improving timely access to childhood cancer diagnosis and treatment. However, because childhood cancer care is considerably more complex than the other PEN-Plus tracer conditions, mid-level facilities will likely retain a predominantly “referral-to-tertiary” function in this domain. This contrasts with other tracer conditions, where PEN-Plus clinics arguably play a more direct role in diagnosis and treatment, rather than serving solely as a referral point to tertiary centres.

### 2.2 Workforce capacity and retention

The aforementioned physical infrastructure challenges are further compounded by a critically thin workforce of paediatric experts. In countries like Liberia, specialized paediatric mentorship and knowledge transfer may rest on a single individual, partly leading to a reliance on providers trained in adult medicine who may treat children as merely “smaller variants” of adults. A paediatric specialist summarized this challenge succinctly;

*“For us, a child is a child, is an entity. But for people who work with adults, a child is half an adult” (Clinician)*

This fragility is further threatened by a lack of staff retention mechanisms; frequent transfers and turnover compromise mentorship strategies and erode the continuity of care and trust that families establish with their providers. This is of particular relevance to chronic disease or disease with long-term sequelae. A program leader highlighted the importance of provider continuity:

*“We need to … reduce turnover of health professionals. It doesn’t look important, but when you change the little thing in your clinic, that patient starts from zero. For you, it’s just someone who changes for the patient. It’s a new life that has to start … the biggest predictor of good care for people, from the patient’s perspective, is the same health provider.” (PEN-Plus implementer)*

### 2.3 Strengthening systems through collateral and off-target benefits

Despite these challenges, building and investing in robust infrastructure for specific conditions has already demonstrated collateral benefits; for instance, sickle-cell disease infrastructure has improved general hematologic diagnosis capacity, while infrastructure for Type I Diabetes has strengthened endocrine care more broadly. To overcome geographic barriers to mentorship, specialists are increasingly using social and digital platforms to scale the reach of their expertise; mid-level training on paediatric conditions may strengthen improve the child health system as a whole. Many of these strategies draw on decades of lessons from HIV and infectious-disease programs, utilizing established hotlines, task-shifting policies, and screening pathways. By shifting stable chronic disease management toward community-based care and peer groups, clinical bandwidth can be reserved for the most complex cases. Therefore, recognition of these ripple effects - both of past investments in vertical (e.g., HIV) programs, as well as the horizontal and vertical off-target effects generated through PEN-Plus will support a stronger investment case for PEN-Plus as a multi-level model for health system strengthening.

## 3 Discussion

*“You need to improve your transfusion services. You need to do pain management and palliative care. You need to look at infection control. So you realize that actually, you went in for one disease, but you are responding to other disease conditions.” (Clinician)*

Hence, the future of PEN-Plus may rely on a deliberate transformation from a specialized model for decentralization to a comprehensive, integrated, systems-based approach. To safeguard paediatric care from being carved out, the region should move beyond the current adult-centric ecosystem by (for example) reforming essential medicine lists, standardizing paediatric diagnostics, and implementing robust staff retention mechanisms to protect its “thin” expert workforce. By integrating the WHO Package of Essential Non-communicable Disease Interventions (PEN) with the PEN-Plus model and leveraging lessons from successful infectious-disease programs, health systems can turn the “off-target” benefits of specialized care into a sustainable standard for all. Ultimately, the goal is to ensure that as PEN-Plus scales, it does not merely expand its reach but extends its commitment to the most vulnerable; scaling-up the comprehensiveness of paediatric care one teachable task at a time.

*“It’s impossible for us to cope with the numbers of patients with chronic diseases coming every three months, every six months … It’s not possible. So we need to find systems that support this expansion of PEN-Plus in ways that we involve the community.” (PEN-Plus champion)*

Therefore, in concluding this perspective, we argue that the next phase of PEN-Plus should be defined not only by expanding access to care but also by strengthening equitable, child-centred, and integrated service delivery within national health systems and building on communities - including carefully extending services to other orphaned conditions like childhood cancer.

## Data Availability

The original contributions presented in the study are included in the article/supplementary material, further inquiries can be directed to the corresponding author/s.
